# Establishing Reference Intervals for Normal Fetal Lung Biological Parameters at 21–40 Weeks of Gestation in the Chinese Population: A Cross-Sectional Study

**DOI:** 10.3390/diagnostics13233525

**Published:** 2023-11-24

**Authors:** Taihui Xia, Shijing Song, Li Wang, Lijuan Sun, Jingjing Wang, Qingqing Wu

**Affiliations:** 1 Department of Ultrasound, Beijing Obstetrics and Gynecology Hospital, Capital Medical University, Beijing 100026, China; xiath1002@163.com (T.X.); dr_songshijing@sina.com (S.S.); wangli1971@ccmu.edu.cn (L.W.); sunlijuan@ccmu.edu.cn (L.S.); wjj_fc@126.com (J.W.); 2 Beijing Maternal and Child Health Care Hospital, Beijing 100026, China

**Keywords:** lung to head ratio, lung area, reference intervals, pulmonary hypoplasia

## Abstract

(1) Background: There is no reliable way to assess antenatal fetal pulmonary hypoplasia; however, the biological parameters of the fetal lung can help in evaluating fetal lung development. This study aimed to establish the reference intervals for normal fetal lung biological parameters at 21–40 weeks among the Chinese population. (2) Methods: This was a cross-sectional study of Chinese groups, and included a total of 1388 normal single pregnant women at 21–40 weeks’ gestation. We selected 2134 images of a standard four-chamber view (4CV). ImageJ software (Release 2.14.0) was used to measure the left and right lung areas using a manual tracing method; the elliptic function key was used to measure the fetal thoracic circumference (TC), thoracic area (TA), head circumference (HC), heart area (HA), and abdominal circumference (AC). Based on the above measurements, the following parameters were calculated: lung area to head circumference ratio (LHR), total lung area (TLA), TLA/Weight (mm^2^/g), cardiothoracic ratio (CTR), lung–thoracic area ratio (TLA/TA), lung–heart area ratio (TLA/HA), TC/AC, and TC/HC. (3) Results: The left and right lung areas and LHRs positively correlated with gestational age (R^2^ = 0.85, 0.88, 0.66, 0.71, *p* < 0.001). From 21–40 weeks, the left and right lung areas and TLA increased by about 3.33 times, 3.16 times, and 3.22 times, respectively. The means of left and right LHRs increased by about 1.94 times and 1.84 times, respectively. TLA/Weight (mm^2^/g) was weakly correlated with gestational age, while CTR, TLA/TA, TLA/HA, TC/AC, and TC/HC had no significant correlation with gestational age. There was no statistically significant difference in fetal lung parameters between different genders of newborns, *p* > 0.05. (4) Conclusions: Our study establishes the reference intervals for normal Chinese fetal lung biological parameters at 21–40 weeks. Moreover, the reference intervals apply to fetuses of different genders. This paper can provide a reference for the prenatal non-invasive assessment of fetal pulmonary hypoplasia.

## 1. Introduction

Pulmonary hypoplasia can lead to severe neonatal respiratory disease (NRDS) and increase infant perinatal mortality. Pulmonary hypoplasia is found in about 7–10% of neonatal autopsies and up to 50% of cases in which other related congenital abnormalities are present. Affecting 9 to 11 of every 10,000 live births, the perinatal mortality is approximately 70% (55–100%) [1,2,3]. Skeletal dysplasias, thoracic mass effect (including congenital diaphragmatic hernia (CDH) and pleural effusions), premature rupture of membranes (PPROM), oligohydramnios, fetal growth restriction (FGR), and congenital heart disease (CHD) with decreased pulmonary blood flow may affect fetal lung development [4,5,6,7]. Prenatal diagnosis of fetal pulmonary hypoplasia has important clinical value for patient consultation and clinical management of obstetrics. However, there is no reliable prenatal method to assess pulmonary hypoplasia [2].

Investigators have attempted to assess fetal lung hypoplasia via two-dimensional ultrasound parameters. Yoshimura S. et al. [8] compared eight different two-dimensional ultrasound parameters of the fetal lungs and the results showed that TC/AC had the best diagnostic accuracy (sensitivity 90.5%, specificity 90.0%) for pulmonary dysplasia. Triebwasser et al. [2] conducted a systematic review of the prediction of pulmonary dysplasia by two-dimensional ultrasound parameters, reporting that TC, TC/AC, and lung area have predictive value for pulmonary dysplasia and that lung area has excellent predictive accuracy. LHR is a commonly used method for assessing fetal prognosis with CDH [9,10]. However, one study has reported [11] that LHR increases with gestational age. Consequently, researchers [12] have further proposed to assess the prognosis of fetuses with CDH via the observed to expected lung area to head circumference ratio (O/E LHR) in order to correct LHR changes with gestational age. O/E LHR has important clinical value in predicting the prognosis of CDH. Previous studies have shown that 2D ultrasound parameters have clinical value in assessing fetal lung hypoplasia.

Peralta et al. established the normal reference values of fetal lung area and LHR at 12–32 weeks’ gestation by including 650 normal fetuses; the current reference values of O/E LHR mainly refer to the results of this study [11,12]. However, the study lacked data on parameters related to fetal lungs after 32 weeks’ gestation. Kehl S. et al. [13] obtained a new formula for calculating LHR by enrolling 126 normal fetuses at 20–40 weeks. Britto [14] established lung area and LHR reference values by including 62 normal fetuses at 20–36 weeks. However, the above studies have the problem of a small sample size at each gestational week; thus, the clinical value of the results needs to be further studied. Larger sample size studies on fetal lung area and O/E LHR reference values after 32 weeks’ gestation are needed.

To establish the normal fetal lung area and LHR reference intervals among the Chinese population at 21–40 weeks gestation, 1388 singleton pregnant women were included in this study and 2143 standard 4CV images were selected, with >100 images per gestational age, providing a reference for the prenatal evaluation of pulmonary hypoplasia.

## 2. Materials and Methods

This cross-sectional study used the images stored in the workstation of the Department of Ultrasound at the Beijing Obstetrics and Gynecology Hospital between December 2017 and October 2022. Inclusion criteria were: (1) singleton pregnancies at 21–40 weeks’ gestation; (2) full term delivery; (3) neonatal Apgar scores of ≥8 at 1, 5, and 10 min after birth, respectively, and no NRDS; (4) gestational age determined according to the last menstrual period and confirmed according to the crown–rump length (CRL) of the fetus at 11–13 + 6 weeks; and (5) measurements of the fetal growth parameters consistent with the gestational age (including biparietal diameter, BPD; HC; AC; femur length, FL). Exclusion criteria were: (1) complications during pregnancy, specifically, gestational diabetes mellitus (GDM), gestational hypertension, or thyroid dysfunction; (2) PPROM; (3) severe oligohydramnios; (4) postpartum neonatal weight > 4000 g or less than the 10th percentile of the corresponding gestational week; (5) fetal structural malformations or chromosomal abnormalities; (6) antenatal corticosteroid (ACS) therapy; or (7) the 4CV did not meet the image quality control standards.

**Figure 1:**
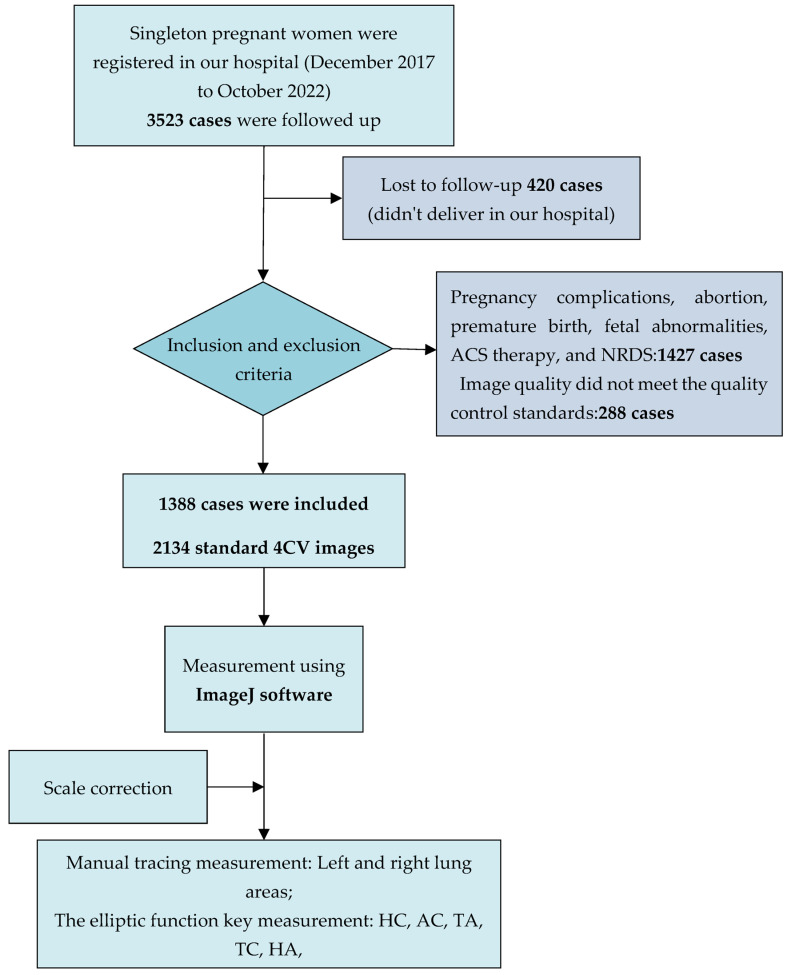
Flowchart of the case enrollment process and parameter measurement for this cross-sectional study. ACS: antenatal corticosteroid; NRDS: neonatal respiratory disease; 4CV: four-chamber view; HC: head circumference; AC: abdominal circumference; TA: thoracic area; TC: thoracic circumference; HA: heart area.

During this study, 3523 singleton pregnancies registered in our hospital were followed up. A total of 420 were excluded due to non-delivery in our hospital; 1427 were excluded due to pregnancy complications, abortion, preterm birth, ACS therapy, fetal abnormalities, and NRDS; and 288 were excluded due to the image quality not meeting the criteria. A total of 1388 cases were included in this study. We selected 2134 standard 4CV images of the fetal heart and measured the biological parameters of the fetal lung. The study flowchart is shown in Figure 1.

**Figure 2:**
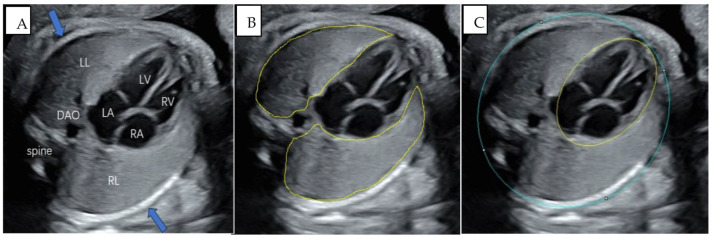
(**A**) Standard 4CV images. The image should show a complete fetal thoracic cage, and the chest walls on both sides can show one complete or nearly complete rib (respectively shown by the arrows). The image should clearly show bilateral lung tissue (LL: left lung, RL: right lung), with the descending aorta (DAO) located in the left front of the fetal spine; the left atrium (LA), right atrium (RA), left ventricle (LV), right ventricle (RV), atrioventricular septum, mitral valve, and tricuspid valve can be shown. (**B**) Measurement of lung areas. Measurement of the area of the left and right lung is conducted using manual tracing. Measurements should be made to include all lung tissue, taking care to avoid the myocardial tissue, large blood vessels, and ribs. (**C**) Measurement of the thoracic circumference (TC), thoracic area (TA), and heart area (HA). Measurements of the TA and TC should include the spine and ribs and should exclude the chest wall soft tissues.

In this study, a 4CV was selected to measure the biological parameters of fetal lungs. The standard image is shown in Figure 2A. Such an image should display a complete fetal thoracic cage, while the chest walls on both sides can show one complete or nearly complete rib (respectively indicated by the arrows in Figure 2A). The image should avoid the ribs and spinal sound shadow to clearly show the left lung and right lung tissues and the descending aorta (DAO) located in the left front of the fetal spine. The left atrium (LA), left ventricle (LV), right atrium (RA), right ventricle (RV), atrioventricular septum, mitral valve, and tricuspid valve should be shown. The images did not contain a measurement caliper or color Doppler. Two sonographers reviewed whether each image met the criteria.

The acquisition of images used seven different ultrasound machines provided by five different manufacturers: including GE Voluson E8/E10 (GE Healthcare Austria GmbH & Co OG, Zipf, Austria), provided with an abdominal curvilinear transducer C1-5-D; SAMSUNG WS80A (Samsung Medison Corporation, Seoul, Republic of Korea), provided with an abdominal curvilinear transducer CA1-7A; PHILIPS EPIQ7/EPIQ7C (Philips Healthcare, Bothell, WA, USA), provided with an abdominal curvilinear transducer C5-1; SIEMENS Acuson S2000 (Siemens Medical Systems, Mountain View, CA, USA), provided with an abdominal curvilinear transducer 9L4; and HI VISION Preirus (Hitachi Aloka Medical, Ltd., Tokyo, Japan), provided with an abdominal curvilinear transducer C715. All images were collected by sonographers in obstetrics and gynecology ultrasound who had more than two years of work experience. To obtain high-quality images, sonographers need to adjust the relevant parameters in real time (such as the gain, depth, frequency, magnification, and time gain compensation) based on the specific conditions used during the examination.

The left and right fetal lung areas were measured separately using the method of manual tracing. Measurements should include all lung tissue, taking care to avoid the myocardial tissue, large blood vessels, and ribs, as shown in Figure 2B. At the same time, the TA, TC, and HA were measured using the ellipse function key. Measurements of the TA and TC should include the spine and ribs and exclude the chest wall soft tissues; HA measurements should include cardiac tissue intact, as shown in Figure 2C. These parameters were measured manually, using retrieved 4CV images. In addition, the HC was measured in the axial view of the fetal head at the level of the thalami, and the AC was measured in the transverse section of the fetal abdomen; the measurement methods were based on the ISUOG Practice Guidelines [15]. The fetal weight was calculated by the Hadlock-3 formula based on biological parameters (HC, AC, FL) [16]. Left/right LHR = left/right lung area (mm^2^)/HC (mm). CTR = TA (mm^2^)/HA (mm^2^). TLA = (left + right) lung areas (mm^2^). All of the above parameters were measured by one sonographer using Image J software (Release 2.14.0, https://imagej.net/software/fiji/, 9 February 2023), with scale correction applied according to the image ruler before measurement. The sonographer was blinded to the actual gestational age.

In 60 arbitrarily selected images (three cases were randomly selected for each gestational age, at 21–40 weeks), the lung areas in the same stored 4CV images were measured using the manual tracing method twice at different times by the same sonographer and once by another sonographer in order to assess the intra- and interobserver agreement.

### Statistical Analysis

Statistical analysis was performed using R software (Version 4.3.1, http://www.R-project.org, 30 July 2023). We established reference intervals for the left and right lung areas and left and right LHRs at 21–40 weeks of gestation. To establish a range of medical reference intervals, we required a sufficiently large sample size of *n* ≥ 100. When establishing the normal reference value range, the single-sample Kolmogorov–Smirnov test was applied to test the normality of the data. If the data conformed to the normal distribution, the reference value range was expressed using the mean value ± 1.96 standard deviations as the upper and lower values, while if it did not conform to a normal distribution, the reference value range was expressed using the percentile method; P2.5 and P97.5 were calculated as the lower and upper values. Spearman correlation analysis and univariate linear regression were used to evaluate the correlation between the fetal lung area and LHR with gestational age, and the regression equation was calculated. Covariance analysis was used to compare whether differences in the fetal lung parameters of different neonatal sexes were statistically significant after correcting for the effect of gestational age. ICC was used to compare the intra- and interobserver measurement agreement, and ICC > 0.75 was considered good. *p* < 0.05 was considered statistically significant.

## 3. Results

### 3.1. Basic Information of the Study Population

A total of 1388 cases were included in this study. We selected 2134 standard 4CV images of the heart and measured the biological parameters of the fetal lung. The basic information for the study population is shown in Table 1.

### 3.2. The Reference Intervals of the Normal Fetal Lung Area and LHR

**Figure 3:**
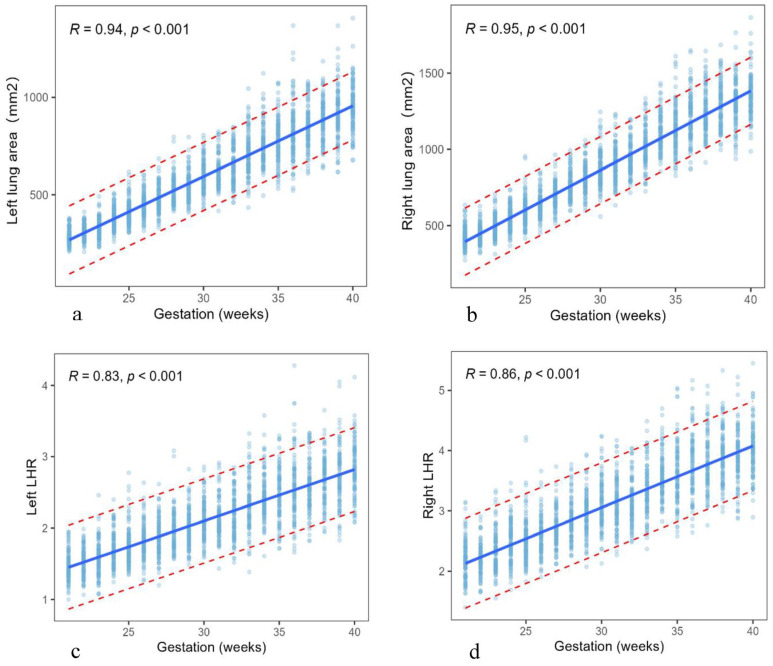
The plot of the left (**a**) and right (**b**) lung areas and left (**c**) and right (**d**) LHRs against gestational age, showing mean and 95% reference intervals (Dotted lines). LHR: lung area to head circumference ratio.

Fetal lung area and LHR increased with gestational age, which was positively correlated with gestational age (left lung area (mm^2^) = −493.57 + 36.25 × GA (wks), R^2^ = 0.85, *p* < 0.001; right lung area (mm^2^) = −698.97 + 52.07 × GA (wks), R^2^ = 0.88, *p* < 0.001; Left LHR = −0.06 + 0.07 × GA (wks), R^2^ = 0.66, *p* < 0.001; Right LHR = −0.02 + 0.10 × GA (wks), R^2^ = 0.71, *p* < 0.001). The relationship between fetal lung area and LHR with gestational age is shown in Table 2 and Figure 3. From 21 to 40 weeks of gestation, the left lung area increased by 665.9 mm^2^ (mean from 285.3 mm^2^ to 951.2 mm^2^), an increase of about 3.33 times, the right lung area increased by 922.9 mm^2^ (mean from 428.1 mm^2^ to 1351.7 mm^2^), an increase of about 3.16 times, and the TLA increased by 1590.5 mm^2^ (mean from 713.4 mm^2^ to 2302.9 mm^2^), an increase of about 3.22 times. The left LHR was about 1.46 (mean) at 21 weeks and was about 2.83 (mean) at 40 weeks, an increase of about 1.94 times. The right LHR was about 2.19 (mean) at 21 weeks and about 4.02 (mean) at 40 weeks, an increase of 1.84 times. The reference intervals of the normal fetal lung area and LHR at 21–40 weeks of gestation are shown in Table 3.

### 3.3. Other Fetal Lung Biological Parameters

**Figure 4:**
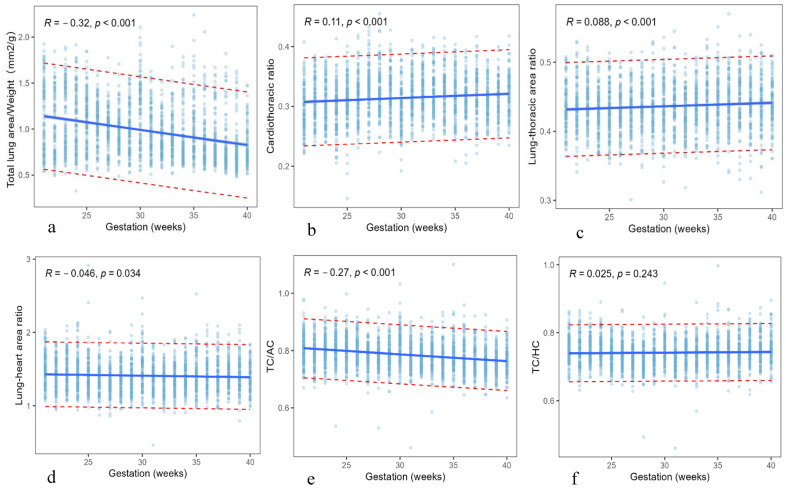
Plot of the biological parameters against gestational age. (**a**) TLA/Weight: total lung area/Weight; (**b**) cardiothoracic ratio; (**c**) TLA/TA: lung–thoracic area ratio; (**d**) TLA/HA: lung–heart area ratio; (**e**) TC/AC; (**f**) TC/HC. TC: Thoracic circumference; AC: Abdominal circumference; HC: Head circumference. Dotted lines indicate 95% reference intervals.

TLA/Weight (mm^2^/g) was weakly correlated with gestational age (R = 0.32, *p* < 0.001), while CTR, TLA/TA, TLA/HA, TC/AC, and TC/HC had no significant correlation with gestational age (R < 0.3). These parameters did not change significantly with gestational age. The relationship between these parameters and gestational age and the range of reference values are shown in Table 4 and Figure 4.

### 3.4. Comparison of Biological Parameters of the Fetal Lung in Different Neonatal Sexes

After excluding the influence of gestational age, there was no statistical significance in the fetal lung biological parameters of different sexes (*p* > 0.05), as shown in Table 5. Therefore, the parameters of the fetal lung are not affected by fetal sex, and there is no need to consider differences in fetal sex when using the reference value of the parameters.

### 3.5. Agreement between Intra-Observer and Inter-Observer Measurements

The ICCs (95% CI) of the intra-observer reproducibility of the left and right lung areas were 0.998 (0.996~0.999, *p* < 0.05) and 0.996 (0.994~0.998, *p* < 0.05), respectively. The ICCs (95% CI) of the inter-observer reproducibility of the left and right lung areas were 0.998 (0.996~0.999) and 0.99 (0.995~0.998), respectively. The ICCs were all greater than 0.75. There was good consistency in the intra-observer and inter-observer measurements.

## 4. Discussion

Our study provided reference intervals of normal Chinese fetal lung biological parameters at 21–40 weeks of gestation. The study data demonstrated that the fetal lung area and LHR increased with gestational age, the lung area increased by about 3.2 times, and the LHR increased by about 1.9 times, from 21 weeks to 40 weeks. However, TLA/Weight (mm^2^/g), CTR, TLA/TA, TLA/HA, TC/AC, and TC/HC were not strongly correlated with gestational age, and the change with gestational age was not apparent. Moreover, the fetal lung biological parameters were not affected by the newborns’ sex.

LHR is an ultrasound indicator most extensively used to evaluate the outcomes in fetuses with CDH; previous studies [17,18] have shown that LHR < 1.0 indicates a poor prognosis. Peralta et al. [11] found that LHR increases with gestational age. Therefore, to exclude the effect of gestational age, certain studies [12,19] have further proposed using O/E LHR to predict postnatal survival in the isolated CDH. Studies have demonstrated that left-sided CDH when O/E LHR ≤ 25% and right-sided CDH when O/E LHR ≤ 45% predict poor outcomes [20]. The European antenatal CDH registry classifies CDH according to O/E LHR: extreme (<15%), severe (15–25%), moderate (26–35%), and mild (36–45%); this criterion applies to isolated left-sided CDH as well [21]. Taking this criterion as the standard, a study group in Toronto reported [22] that survival rates were approximately 0%, 20%, 30% to 60%, and >75% for extreme, severe, moderate, and mild, respectively. The current reference values of the fetal lung area and the O/E LHR are mainly derived from a study of 650 normal fetal lung evaluations at 12–32 weeks of gestation [11]. This study established the reference value of the normal fetal LHR in the Chinese population at 21–40 weeks of gestation, making up for the lack of data after 32 weeks for the previous reference value and allowing the applicable population to be better targeted.

Long-term PPROM causes severe oligohydramnios, which affects the development of fetal lungs, resulting in pulmonary hypoplasia; prediction of pulmonary hypoplasia is important for optimal management [3,23]. Triebwasser et al. [2] conducted a systematic review of the value of two-dimensional ultrasound parameters in the evaluation of pulmonary hypoplasia, reporting that the TC, TC/AC, and lung area have clinical value in the diagnosis of pulmonary hypoplasia and that the lung area has excellent predictive accuracy (sensitivity 78–81%, specificity 75–100%). The Aafreen S [24] and Achiron R [25] studies reported that the right lung area was significantly smaller in patients with pulmonary dysplasia. Therefore, it is of great clinical value to establish the reference values of the lung area in the Chinese population. Vintzileos et al. [26] compared the accuracy of six different fetal lung parameters in predicting fatal fetal pulmonary dysplasia, among which (CA − HA) × 100/CA had the best diagnostic accuracy (sensitivity 85%, specificity 85%). (CA − HA) × 100/CA has similarities with TLA/TA in our study, which excluded the area of surrounding tissues and ribs and is better than (CA − HA) × 100/CA for reflecting lung development. Yoshimura S et al. [8] compared eight different ultrasonographic parameters, and their results showed that TC/AC had the best diagnostic accuracy (sensitivity 90.5%, specificity 90.0%). The same study reported that TC/AC and LA (lung area)/TA did not change with gestational age, which is consistent with our findings. The value of TC/AC in the study of Yoshimura S [8] was 0.88 ± 0.044, which is greater than our study’s TC/AC: 0.78 (0.69, 0.90), while the LA/TA (0.40 ± 0.05) is similar to the results of our study (TLA/TA: 0.44 (0.37, 0.50)).

Recently, animal experiments [6] have shown that CHD with decreased pulmonary blood flow caused the lungs of neonatal rats to become smaller and decreased the number of type 2 alveolar cells, resulting in lung hypoplasia. Other studies [27,28] have reported that FGR can impair fetal lung function, as depicted by higher parenchymal resistance, and increase the incidence of neonatal respiratory diseases. Few studies have reported prenatal evaluation of fetal lung development in FGR and CHD with decreased pulmonary blood flow. Therefore, establishing the reference ranges of normal fetal lung biological parameters at different gestational weeks can provide a reference basis for the assessment of fetal lung development in such patients.

Peralta’s study [11] reported lung area and LHR reference values at 12–32 weeks and their calculation equations. The fetal lung area and LHR reported by Peralta at 21–32 weeks were slightly smaller than those reported in this study. There are significant differences in the formulas used to calculate lung area and LHR compared with our study, possibly due to the different gestational age ranges included. Therefore, Peralta’s formulas may not fit the calculation of the expected LHR at all gestational weeks. Kehl S [13] enrolled 124 normal fetuses at 20–40 weeks of gestation and established new formulas for calculating the LHR: Right LHR = −4.7290746 + 1.600050 × GA1⁄2 (wk) and Left LHR = −5.646511 + 2.751560 × GA1⁄3 (wk). This result considered the LHRs to be linearly associated with gestational age, similar to our result. However, the LHR values calculated by these formulas are significantly different from those obtained in our study. The sample size of the study was small; thus, the clinical value of the results needs to be further verified. Britto et al. [14] established the reference values of fetal lung parameters at 20–36 weeks of pregnancy by performing 214 ultrasound tests on 62 healthy fetuses. The reported lung area and LHR values were larger than in our findings. Their lung–head ratio calculation formula (Right LHR = −0.3026 + 0.05259 × MA, Left LHR = −0.6213 + 0.05072 × MA) and this study’s obtained formula were both linear regression equations (Right LHR = −0.02 + 0.10 × GA, Left LHR = −0.06 + 0.07 × GA).

This study has a number of limitations. First, this study was retrospective, using 4CV of the fetal heart, HC, and AC measurements from cross-section images which were stored in an ultrasound workstation and acquired by different sonographers using different ultrasound instruments. Therefore, the images were selected in strict accordance with quality control criteria and evaluated by two sonographers. Obtaining these images from different ultrasound devices did not affect the actual area measurement of the fetal lungs. Second, the types of 4CV images used in this study were not uniform, as there is no uniform standard to determine which type of 4CV is the most accurate for lung measurement.

The value of the fetal lung area and the LHR in the assessment of fetal lung development in FGR and CHD with decreased pulmonary blood flow can be a new research direction.

## 5. Conclusions

In our study, fetal lung area and LHR increased with gestational age and were positively correlated with gestational age. TLA/Weight (mm^2^/g) was weakly negatively correlated with gestational age, while CTR, TLA/TA, TLA/HA, TC/AC, and TC/HC had no significant change with gestational age. The intra-observer and inter-observer reproducibility for left and right lung area measurements were good. We established the reference intervals of normal Chinese fetal lung biological parameters at 21–40 weeks, in line with the national conditions. These results can provide a reference for the prenatal non-invasive assessment of fetal pulmonary hypoplasia. There was no statistically significant difference in fetal lung parameters between different sexes of newborns; thus, the reference interval applies to fetuses of different sexes.

## Figures and Tables

**Table 1:** Basic characteristics of the study population.

Variable	Mean ± SD or N (%)
Maternal age, y	31.1 ± 3.9
Gestational age at delivery, Weeks	39.2 ± 1
Cesarean delivery/Vaginal delivery	390 (28.1%)/998 (71.9%)
Neonatal gender (male/female)	729 (52.5%)/659 (47.5%)
Neonatal weight, g	3348.4 ± 358.6
Neonatal height, cm	50 ± 1.5

**Table 2:** Regression equations for the left and right lung area and LHR to gestational age.

	Regression Equations	R^2^	*p*
Left lung area	−493.57 + 36.25 × GA (wks)	0.85	<0.001
Right lung area	−698.97 + 52.07 × GA (wks)	0.88	<0.001
Left LHR	−0.06 + 0.07 × GA (wks)	0.66	<0.001
Right LHR	−0.02 + 0.10 × GA (wks)	0.71	<0.001

LHR: lung area to head circumference ratio; GA: gestational age.

**Table 3:** Lung area (manual tracing) and LHR from 21 to 40 gestational weeks.

GA (wks)	N	Mean (95% Reference Interval) (Areas: mm^2^)				
		Left Lung AREA	Left LHR	Right Lung Area	Right LHR	TLA (Left + Right)
21	113	285.3 (218.7, 372.2)	1.46 (1.14, 1.91)	428.1 (322.1, 593.5)	2.19 (1.69, 2.87)	713.4 (564.1, 928.7)
22	102	309.4 (237.3, 379.3)	1.53 (1.19, 1.87)	445.7 (341.8, 573.1)	2.20 (1.67, 2.73)	755.2 (598.2, 913.1)
23	111	327.5 (235.1, 441.6)	1.57 (1.17, 2.02)	485.1 (384.1, 663.1)	2.33 (1.82, 3.15)	812.7 (650.8, 1067.1)
24	112	377.3 (297.4, 484)	1.7 (1.36, 2.17)	548.8 (413.8, 682.3)	2.48 (1.91, 3.03)	926.1 (750.2, 1127.1)
25	127	409.4 (313.3, 547.6)	1.74 (1.4, 2.39)	591.7 (455.6, 775.5)	2.52 (2.01, 3.4)	1001.1 (791.3, 1272.3)
26	113	447.2 (341.3, 575.3)	1.82 (1.41, 2.33)	635.2 (503.9, 812)	2.58 (2.03, 3.24)	1082.4 (884.2, 1330.5)
27	106	478.2 (361.3, 601.4)	1.87 (1.44, 2.4)	691 (547.6, 859.2)	2.70 (2.19, 3.26)	1169.2 (945.2, 1449.2)
28	105	522.9 (429.9, 651.8)	1.96 (1.6, 2.38)	760.2 (636.4, 954)	2.84 (2.32, 3.62)	1283 (1089.1, 1568.6)
29	102	548.1 (430.1, 693.3)	2.00 (1.58, 2.52)	811.1 (639.2, 1021.1)	2.95 (2.32, 3.66)	1359.2 (1087.2, 1662.4)
30	108	594.8 (454.3, 757.7)	2.11 (1.64, 2.66)	878.1 (690.8, 1148.5)	3.12 (2.48, 4.00)	1472.9 (1195.8, 1840.8)
31	105	616.3 (516.3, 809.6)	2.13 (1.75, 2.78)	906 (717, 1183.5)	3.13 (2.48, 4.01)	1522.2 (1266.2, 1894.4)
32	108	643.8 (488.8, 815.3)	2.17 (1.66, 2.76)	935.7 (786.9, 1160.4)	3.15 (2.58, 3.96)	1579.5 (1293.1, 1903.6)
33	103	714.5 (514.1, 927.9)	2.34 (1.67, 3.08)	1014.4 (774.8, 1296.6)	3.33 (2.63, 4.13)	1728.9 (1308.4, 2147.1)
34	104	759.7 (623.4, 992.7)	2.44 (1.95, 3.11)	1110 (935.9, 1368.9)	3.57 (3.01, 4.34)	1869.7 (1564.7, 2270)
35	104	782.2 (571.1, 1043.2)	2.47 (1.82, 3.24)	1138.8 (879, 1549.9)	3.59 (2.74, 4.90)	1921 (1532.2, 2433.3)
36	103	826.4 (593.8, 1134.4)	2.56 (1.81, 3.59)	1215.5 (943.9, 1510.9)	3.76 (2.92, 4.70)	2041.9 (1543.6, 2611)
37	102	850.3 (642.3, 1065.7)	2.59 (1.95, 3.3)	1246 (1028.5, 1540.4)	3.80 (3.09, 4.71)	2096.4 (1727.5, 2542.1)
38	103	885.2 (680.6, 1080.2)	2.68 (2.06, 3.27)	1292 (1065.6, 1572)	3.92 (3.20, 4.72)	2177.2 (1778.2, 2607.6)
39	101	909.7 (663.5, 1187.4)	2.72 (1.97, 3.49)	1299.8 (1003.7, 1550.9)	3.89 (2.99, 4.77)	2209.6 (1674.5, 2658.2)
40	102	951.2 (742.8, 1149.3)	2.83 (2.15, 3.48)	1351.7 (1122.5, 1665)	4.02 (3.33, 4.92)	2302.9 (1889.5, 2767.4)

GA: gestational age; N: number; LHR: lung area to head circumference ratio; TLA: total lung area.

**Table 4:** The mean values (95% reference interval) of parameters at 21–40 weeks.

Parameters	Mean (95% Reference Interval)	R	*p*
TLA/Weight (mm^2^/g)	0.99 (0.57,1.67)	−0.32	<0.001
CTR	0.31 (0.25, 0.39)	0.11	<0.001
TLA/TA	0.44 (0.37, 0.50)	0.088	<0.001
TLA/HA	1.41 (1.04, 1.89)	−0.046	0.034
TC/AC	0.78 (0.69, 0.90)	−0.27	<0.001
TC/HC	0.74 (0.67, 0.83)	0.025	0.243

TLA: Total lung area; TC: Thoracic circumference; TA: Thoracic area; HA: Heart area; HC: Head circumference; AC: Abdominal circumference; CTR: Cardiothoracic ratio (TA/HA).

**Table 5:** Comparison of biological parameters of the fetal lung in different neonatal sexes.

Parameters	Median (First Quartile, Third Quartile)			
	Male	Female	F	*p*
Left lung area (mm^2^)	574.1 (419.3, 777.8)	581.7 (411.2, 785.2)	0.002	0.969
Right lung area (mm^2^)	851.9 (603.5, 1145.0)	871.3 (601.6, 1134.5)	0.939	0.333
TLA (left + right) (mm^2^)	1430.9 (1032.8, 1919.4)	1457.5 (1023.6, 1914.9)	0.232	0.630
Left LHR	2.07 (1.74, 2.45)	2.04 (1.72, 2.50)	0.121	0.729
Right LHR	3.01 (2.53, 3.58)	3.04 (2.54, 3.60)	0.186	0.666
TLA/Weight (mm^2^/g)	0.93 (0.74, 1.20)	0.89 (0.73, 1.20)	0.090	0.764
CTR	0.31 (0.29, 0.34)	0.31 (0.29, 0.34)	0.012	0.912
TLA/TA	0.44 (0.41, 0.46)	0.44 (0.41, 0.46)	0.814	0.367
TLA/HA	1.39 (1.25, 1.55)	1.39 (1.26, 1.56)	0.417	0.519
TC/AC	0.78 (0.75, 0.82)	0.78 (0.75, 0.82)	0.005	0.943
TC/HC	0.74 (0.71, 0.77)	0.74 (0.71, 0.77)	1.759	0.185

LHR: Lung area to head circumference ratio; TLA: Total lung area; TC: Thoracic circumference; TA: Thoracic area; HA: Heart area; HC: Head circumference; AC: Abdominal circumference; CTR: Cardiothoracic ratio (TA/HA).

## Data Availability

The datasets used and/or analyzed during the current study are available from the corresponding author on reasonable request.

## Abbreviations

4CV: Four-chamber view; TC: Thoracic circumference; TA: Thoracic area; HC: Head circumference; HA: Heart area; AC: Abdominal circumference; LHR: Lung area to head circumference ratio; TLA: Total lung area; CTR: Cardiothoracic ratio; TLA/TA: Lung-thoracic area ratio; TLA/HA: Lung-heart area ratio; NRDS: Neonatal respiratory disease; CDH: Congenital diaphragmatic hernia; PPROM: Premature rupture of membranes; FGR: Fetal growth restriction; CHD: Congenital heart disease; O/E LHR: Observed to expected lung area to head circumference ratio; CRL: crown-rump length; BPD: Biparietal diameter; FL: Femur length; GDM: Gestational diabetes mellitus; ACS: Antenatal corticosteroid; LA: Left atrium; LV: Left ventricle; RA: Right atrium; RV: Right ventricle; LLA: Left lung area; RLA: Right lung area; DAO: Descending aorta; CA: Chest area; MA: Menstrual age; GA: gestational age.
